# Effects of a Workplace Exercise Program on Stress, Burnout, and Quality of Life in Radiologic Technologists: A Randomized Controlled Trial

**DOI:** 10.3390/healthcare14081063

**Published:** 2026-04-16

**Authors:** Pedro Ramalho, António Nunes, Fernanda M. Silva, André Ramalho, Gonçalo Flores, Diogo Monteiro, Pedro Duarte-Mendes

**Affiliations:** 1 Department of Management and Economics, University of Beira Interior (UBI), 6201-001 Covilhã, Portugal; ramalho.pedro@sapo.pt (P.R.); anunes@ubi.pt (A.N.); 2 ULS Castelo Branco, Amato Lusitano Hospital, 6000-085 Castelo Branco, Portugal; 3 NECE—Research Center for Business Sciences, 6200-209 Covilhã, Portugal; 4 School of Education and Communication, University of Algarve, 8005-139 Faro, Portugal; fmadsilva@ualg.pt; 5 CIPER, Faculty of Sport Sciences and Physical Education, University of Coimbra, 3004-531 Coimbra, Portugal; 6 Sport Physical Activity and Health Research and Innovation Center, SPRINT, 6000-084 Castelo Branco, Portugal; andre.ramalho@ipcb.pt; 7 Department of Sports and Well Being, Polytechnic Institute of Castelo Branco, 6000-084 Castelo Branco, Portugal; 8 Faculty of Sport, University of Porto, 4099-022 Porto, Portugal; goncalofloresft@outlook.com; 9 ESECS, Polytechnic University of Leiria, 2411-901 Leiria, Portugal; diogo.monteiro@ipleiria.pt; 10 Research Center in Sports Sciences, Health Sciences, and Human Development (CIDESD), 2411-091 Leiria, Portugal

**Keywords:** health-related exercise, well-being, burnout syndrome, quality of life, radiologic technologists

## Abstract

Background/Objectives: Radiologic technologists are frequently exposed to occupational stressors that heighten the risk of burnout, compromising well-being and job performance. Workplace exercise programs have been identified as promising strategies to enhance physical and mental health across occupational groups; however, robust experimental evidence among radiologic technologists remains limited. This study aimed to evaluate the effects of a structured workplace exercise program on perceived stress, burnout, and quality of life among radiologic technologists. Methods: A small-scale randomized controlled experimental study was conducted with 19 radiologic technologists from the Local Health Unit of Castelo Branco, Portugal. Participants were randomly assigned to an experimental group (*n* = 10, age mean = 43.8 ± 9.92 years old) or a control group (*n* = 9, age mean = 48.2 ± 7.86 years old). The intervention consisted of a six-week workplace exercise program conducted during work hours, comprising sessions three times per week, twice per day. Each session lasted approximately 15–20 min and included balance, stretching, and light resistance exercises. Outcomes were assessed pre- and post-intervention using the Perceived Stress Scale, the Copenhagen Burnout Inventory, and the WHOQOL-BREF. Results: The experimental group showed significant reductions in perceived stress (*p* = 0.013, *d =* −0.697 (−1.6–0.206) [moderate]) and in personal (*p* = 0.004, *d =* −0.834 (−1.748–0.08) [moderate]) and work-related burnout (*p* = 0.026, *d =* −0.756 (−1.664–0.151) [moderate]), as well as improvements in the physical (*p* = 0.046, *d =* 0.592 (−0.303–1.488) [small]) and environmental (*p* = 0.032, *d =* 0.991 (0.062–1.92) [moderate]) domains of quality of life. No significant changes occurred in the control group. Conclusions: These preliminary findings suggest that a brief, low-cost workplace exercise program may reduce stress and burnout and improve quality of life among radiologic technologists. These findings support the integration of structured physical activity into healthcare work settings as a feasible, preventive, and health-promoting strategy.

## 1. Introduction

Healthcare professionals are continually exposed to demanding, stressful work environments that can compromise both their physical and mental health. Among them, radiologic technologists face unique occupational challenges, including high workloads, exposure to ionizing radiation, and prolonged static postures, which contribute to increased stress and burnout [1]. According to the Job Demands–Resources theoretical model, high work demands, such as cognitive load and technical complexity, when not balanced by adequate organizational resources, progressively lead to burnout and loss of professional fulfillment [2,3]. Evidence shows that in health care workers burnout is associated with high levels of cognitive demand, organizational pressure, institutional support, and lower job satisfaction [4,5]. Recent studies reveal that burnout is highly prevalent among radiographers, with 56.5% reporting high emotional exhaustion, 31.5% experiencing high depersonalization, and 64.8% low personal accomplishment, while 24.1% present overall high burnout [1]. Burnout syndrome, characterized by emotional exhaustion, depersonalization, and reduced personal accomplishment, has become a critical concern in healthcare, affecting job satisfaction, performance, and overall quality of life [6]. The World Health Organization [7] has recognized burnout as an occupational phenomenon resulting from chronic workplace stress that has not been successfully managed, underlining its public health relevance. In addition, it has been shown that the volume and complexity of the cases attended, the cognitive demand and the emotional load of the work are relevant determinants of burnout compared to the absolute number of working hours [8,9].

The promotion of workplace health through physical activity has gained increasing attention as a preventive and restorative strategy. In general occupational settings, several studies have shown that regular physical exercise helps to reduce perceived stress, enhance psychological well-being, and improve job satisfaction, being particularly effective among office-based and sedentary workers, where it counteracts the adverse effects of inactivity and postural strain [10,11]. However, similar benefits have been observed within a more demanding context characterized by high physical and emotional workloads, shift work, and exposure to psychosocial stressors. Evidence in this population suggests that regular physical activity is associated with lower levels of stress and burnout symptoms and greater psychological well-being and quality of life [12]. Despite these promising findings, the implementation of structured exercise programs in hospital environments remains limited. Specifically, the available literature on radiology technicians, who face distinct ergonomic and psychological challenges, remains scarce and fragmented, highlighting a discipline-specific gap in understanding the applicability and effectiveness of workplace physical activity within this professional group.

The current research field presents divergent perspectives regarding the effectiveness and duration of workplace exercise programs. Some studies report significant improvements in well-being, musculoskeletal comfort, and job satisfaction following short interventions among health professionals [13,14]. In contrast, others argue that the effects are modest or short-lived without organizational support and behavioral reinforcement [15]. Recent evidence indicates that short-term workplace exercise interventions, particularly those lasting around six weeks, can produce meaningful physical and psychological benefits among healthcare workers. Studies using semi-supervised programs with two to three sessions per week, lasting 60–80 min per session, have shown significant reductions in stress, anxiety, and depressive symptoms, along with improvements in physical fitness and perceived well-being [16]. This ongoing debate underscores the need for robust experimental evidence to clarify the extent and mechanisms by which physical activity at work influences psychological and quality-of-life outcomes in healthcare settings.

Recent reviews highlight that most studies on burnout and physical activity in the healthcare work environment focus on physicians and nurses, and there is a lack of intervention studies specifically aimed at radiology technicians [4,17,18]. In addition, other studies show that investigations have been mostly observational, lacking studies that test preventive interventions in radiology technicians [8,19]. In this sense, the gap that the present study intends to fill consists of evaluating the effectiveness of a structured physical exercise program in the workplace, developed for radiology technicians, since they are a professional group with exposure to psychosocial risk factors [20].

As there are few experimental studies specifically in radiology technicians, this study aimed to evaluate the effects of a six-week structured workplace exercise program on perceived stress, burnout, and quality of life among radiologic technologists. By adopting a randomized controlled design, the study sought to provide empirical evidence on the feasibility and effectiveness of integrating brief, structured exercise sessions into the routines of healthcare workers. The exercise program is primarily designed to improve the stress levels, burnout, and quality of life of radiology technicians. To address these objectives with methodological rigor, the following section outlines the study design, participant selection, and experimental procedures used to evaluate the impact of the workplace exercise program on radiologic technologists.

## 2. Materials and Methods

### 2.1. Study Design

This was a single-center, parallel-group, two-arm randomized controlled trial with a 1:1 allocation ratio. Participants were assessed at baseline and after six weeks of follow-up. This clinical trial was registered at ClinicalTrials.gov under the identification number NCT07269834 (date of registration: 25 November 2025), ensuring transparency and adherence to international standards for the registration and reporting of randomized controlled trials. Participant recruitment and baseline assessments began in August 2025, followed by a six-week workplace exercise intervention. Primary completion occurred in October 2025, and study completion occurred in December 2025. The manuscript was reported in accordance with the Consolidated Standards of Reporting Trials (CONSORT) guidelines.

### 2.2. Participants

In this study, the target population included healthy radiographers (diagnostic and therapeutic imaging technologists). The sample consisted of professionals from the Local Health Unit of Castelo Branco (ULSCB) who agreed to participate in the study. Participants who met all inclusion criteria (being radiographers and in good health) were randomly assigned to two parallel groups—an experimental group (*n* = 10) and a control group (*n* = 9), using a 1:1 allocation ratio.

The randomization process was conducted using a computer-generated random number sequence, under the supervision of an independent researcher who was not involved in participant recruitment or intervention delivery. Allocation concealment was ensured by using sealed, opaque envelopes prepared in advance. The principal investigator enrolled participants, and group allocation was revealed only after enrollment in accordance with the concealed randomization sequence. Due to the nature of the intervention, neither the participants nor the principal investigator could be blinded to group allocation. Outcome data were collected using self-administered, paper-based questionnaires distributed to radiographers of the ULSCB. Participants were instructed not to disclose their group allocation during outcome assessment. Data were anonymized before analysis, and statistical analyses were conducted by a researcher blinded to group allocation.

The final sample comprised 19 radiographers, with a mean age of 45.89 years (SD = 9.27). A total of 19 eligible participants were selected and randomized, with the entire initial sample retained, as there were no dropouts during the six-week intervention period (experimental group, *n* = 10, age mean = 43.8 ± 9.92 years old; control group, *n* = 9, age mean = 48.2 ± 7.86 years old). Participants reported working an average of 35 h per week in their regular professional activities.

The professional experience of radiologic technologists was considered in the sample characterization. The experimental group had a mean professional experience of 22.8 ± 10.5 years, and the control group had a mean of 27.2 ± 8.3 years. Overall, participants presented a mean of 24.9 ± 9.5 of professional experience.

**Figure 1:**
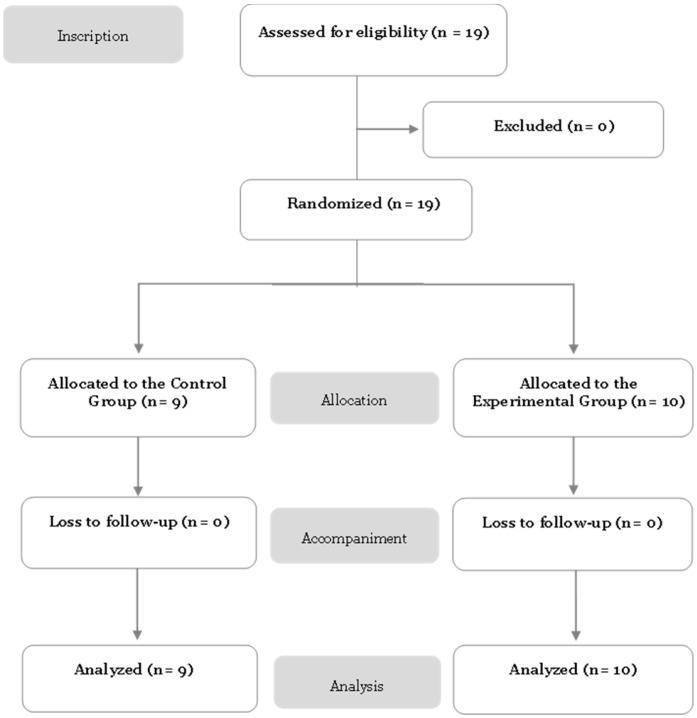
Attendee registration, allocation, and analysis flowchart.

The CONSORT flow diagram is presented in Figure 1.

After formal authorization and a favorable opinion from the Ethics Committee of the Local Health Unit of Castelo Branco (ULSCB), the research began with the distribution of the respective informed consent forms and questionnaires. In preparing the study, the fundamental principles of bioethics were respected, i.e., autonomy, non-maleficence, beneficence, and justice.

Informed consent was obtained from all participants, ensuring anonymity, data confidentiality, and use for scientific purposes only. Participants were informed that no financial compensation would be provided and that they could withdraw at any time without any consequences.

Authorization was requested and obtained from the authors of the scales and questionnaires used in this study (Perceived Stress Scale (PSS), Copenhagen Burnout Inventory (CBI), and WHOQOL-BREF). The questionnaire was designed to avoid any discomfort.

### 2.3. Data Collection Procedure

The data were collected at two distinct time points: before (pre-test) and after (post-test) the intervention. The instruments applied included questionnaires designed to assess the dependent variables defined for the study.

Data were collected directly from participants through a questionnaire, ensuring consistent administration conditions for both groups. After gathering the data, the results were recorded in Excel and checked to maintain the accuracy and integrity of the information.

Only data from participants who completed all study stages were included in the analysis; incomplete or inconsistent responses were excluded. This step ensured the internal validity of the research, enabling the results to more accurately reflect the intervention’s effects.

Data collection was carried out using a paper-based questionnaire. According to Fortin [21], this kind of instrument aims to collect factual data about participants’ feelings and opinions under specific circumstances. The questionnaire opened with a brief explanation of the study’s purpose and assured confidentiality and anonymity throughout the process.

#### Data Collection Tools

The questionnaire was divided into four sections:

First Section—Sociodemographic Variables: This section describes the sample based on sociodemographic factors. It includes 41 multiple-choice or short-answer questions designed to assess variables such as gender, age, weight, height, educational level, work schedule type, number of daily working hours, time spent seated during work, marital status, medical history, smoking and alcohol habits, and physical activity levels.

Second Section—Quality of Life (WHOQOL-BREF): The World Health Organization Quality of Life—BREF (WHOQOL-BREF) was used in this section. The Portuguese version was adapted and validated by Canavarro et al. [22]. The WHOQOL-BREF consists of 26 items distributed across four domains: physical health, psychological well-being, social relationships, and environment. Items are rated on a 5-point Likert scale, where higher scores indicate a better perception of quality of life. In addition to the four domains, the instrument includes two general questions assessing overall perceptions of quality of life and health. Scoring follows the World Health Organization guidelines, with domain scores transformed to a 0–100 scale, enabling comparisons across domains and populations. The WHOQOL-BREF demonstrates strong psychometric properties, including good validity and reliability, and has been adapted and validated for the Portuguese population.

Third Section—Burnout (CBI): The Copenhagen Burnout Inventory (CBI), developed by Kristensen et al. [23] and adapted for the Portuguese population by Fonte [24], was used to assess burnout levels. The instrument contains 19 items divided into three subscales: Personal Burnout (assesses the level of general physical and psychological exhaustion and fatigue experienced by the individual (6 items)); Work-Related Burnout (measures the physical and psychological fatigue perceived as being associated with one’s work (7 items)).

Client-Related Burnout (measures physical and psychological fatigue associated with direct contact with clients or patients (6 items)). The frequency of these feelings is rated on a five-point scale from 0 to 100. Scores of 50 or higher indicate high levels of burnout [23].

Fourth Section—Stress (PSS): The Perceived Stress Scale (PSS) is one of the most widely used tools for assessing perceived stress. It was developed by Cohen, Kamarck, and Mermelstein [25] and aims to measure an individual’s subjective perception of stress intensity in their life, specifically, the degree to which situations are evaluated as unpredictable, uncontrollable, or overwhelming. The Portuguese version of the PSS-10 was validated by Trigo et al. [26]. The frequency of feelings is rated on a five-point scale, ranging from 0 (“never”) to 4 (“very often”).

### 2.4. Work Exercise Program

Participants participated in a six-week workplace exercise program consisting of structured sessions based on methodologies described in previous studies [27,28]. Sessions were held twice daily, three times per week, and lasted approximately 15 to 20 min each.

Each session started with a warm-up, followed by 20 stretching, balance, and strengthening exercises targeting the main body areas: neck, upper limbs, lower back, and lower limbs. Participants performed one to two sets of each exercise for the primary muscle–tendon groups, holding each stretch for 10 to 15 s in a controlled manner until they felt tension or slight discomfort—avoiding any sudden or painful movements—according to the recommendations of the American College of Sports Medicine [28].

The sessions took place at the workplace, during one of the radiographers’ regular breaks, to incorporate the program into the daily work routine without disrupting normal service operations. Each participant did their own exercise program during their break. They each had approximately 90–120 min of exercise per week. Adherence to the program was monitored throughout the six weeks and recorded as a percentage of the total number of sessions attended by each participant. For analytical purposes, only participants with an adherence rate of 80% or higher to the exercise program were included in the final analysis. Those who did not meet this criterion were excluded. Members of the research team supervised the initial sessions to ensure the exercises were performed correctly. Exercise explanations were provided by qualified professionals. In subsequent sessions, autonomous practice was encouraged, supported by illustrated handouts containing detailed instructions. Additionally, weekly reminders were sent to participants via private message to promote continued adherence.

Each training session consisted of three phases:

- Warm-up (1–2 min);
- Main phase (10–12 min);
- Cool-down (1–2 min).

The main phase included predominantly standing exercises aimed at improving:

- Flexibility (static and dynamic exercises; 3×/week; 1–2 sets per exercise; 10–15 s each);
- Balance (static and dynamic exercises; 3×/week; 1–2 sets per exercise; 10–20 repetitions; 3–6 m);
- Strength (bodyweight and resistance exercises using auxiliary equipment; 3×/week; 1–2 sets per exercise; 10–15 repetitions).

The training load was progressively increased every two weeks over the six-week intervention, following American College of Sports Medicine recommendations [28,29]. The detailed progression of load characteristics for flexibility, balance, and strength exercises over the six weeks is presented in Table 1, and the complete exercise program is provided in File S1.

Auxiliary equipment included a stable chair with a backrest for lumbar support, a 1.5 m elastic band, and a 35 cm elastic loop band, both of medium resistance and light blue.

### 2.5. Data Analysis Procedure

Ribeiro [30] highlights that data analysis procedures can be classified in different ways depending on the research priorities, emphasizing that statistical analysis is intrinsically linked to inductive methods, particularly within an empirical approach.

#### 2.5.1. Preliminary Analysis

Data verification revealed no missing values and no outliers, as assessed through visual inspection of boxplots and standardized z-scores. The sample size was calculated a priori using G*Power (version 3.1.9.2, University of Kiel, Germany) [31]. For within-group comparisons (pre-test vs. post-test), the test family was defined as *t*-tests, and the statistical test selected was the paired–samples *t*-test. Assuming an effect size (d) = 0.9, α = 0.05, and power (1–β) = 0.8, a minimum of 9 participants was required, which was achieved in this study. For between-group comparisons (experimental vs. control), the test family was also defined as *t*-tests, and the statistical test for two independent means (two groups) was used. Assuming effect size (d) = 0.9, α = 0.05, and power (1–β) = 0.5, a minimum of 16 participants (8 per group) was required, which was also met.

#### 2.5.2. Main Analysis

Data analysis was conducted using IBM SPSS Statistics, version 30.0 (IBM Corp., Chicago, IL, USA). Descriptive statistics (mean ± standard deviation) were calculated for all continuous study variables, while categorical variables were expressed as counts and percentages. The homogeneity of variances was tested with Levene’s test (*p* < 0.05 indicating rejection of the null hypothesis of equal variances), and the normality of distributions was evaluated using the Shapiro–Wilk test. To assess within-group differences between pre- and post-test measurements, paired *t*-tests and Wilcoxon signed-rank tests were employed. Conversely, independent *t*-tests and Mann–Whitney U tests were utilized to compare between-group differences at both time points. For categorical variables, Fisher’s exact test was applied due to the small sample size.

Additionally, magnitude-based inferences were computed using the following effect size thresholds [32]: 0–0.2: trivial; 0.21–0.6: small; 0.61–1.2: moderate; 1.21–2.0: large; >2.0: very large. GraphPad Prism 9.0 (GraphPad Software, San Diego, CA, USA) was used for data visualization and graphical representation. The interpretation of statistical tests was based on a significance level of *p* ≤ 0.05.

## 3. Results

Table 2 presents the baseline characteristics of all participants and then separated by groups (control vs. experimental). There were no significant differences between groups at baseline (*p* > 0.05) for sociodemographic characteristics. No adverse events associated with the intervention program or procedures were reported. The average adherence to the exercise program was 96.66%. The quantitative data analysis allowed for the evaluation of variations in perceived stress, burnout, and quality of life among radiographers between the baseline (M1) and follow-up (M2), comparing the experimental group, which participated in the workplace exercise program, with the control group, which maintained its usual work routine.

The tables summarizing the results for the experimental groups, as described below, are available in Table 3 and Table 4, respectively.

At both measurement points, no significant differences were found between groups (*p* > 0.05). However, in the experimental group, significant improvements were observed in several dimensions after the intervention. Regarding quality of life, statistically significant differences were observed in the physical domain (M1 = 67.86 ± 12.82; M2 = 74.64 ± 9.88; *p* = 0.046), with a small effect size (d = 0.592), and the environmental domain (M1 = 64.07 ± 7.97; M2 = 70.94 ± 5.71; *p* = 0.032), with a moderate effect size (d = 0.991). The psychological domain showed a trend toward significance (*p* = 0.062), with increased mean values (from 60.00 to 62.92) and a large effect size (d = 1.462), suggesting improved emotional well-being. Overall, the results indicate a global increase in quality of life following the implementation of the workplace exercise program.

In relation to burnout, significant reductions were recorded in the personal (M1 = 52.08 ± 17.92; M2 = 37.08 ± 18.05; *p* = 0.004, *d =* −0.834), work-related (M1 = 48.57 ± 13.60; M2 = 36.07 ± 19.01; *p* = 0.026, *d =* −0.756), and total index (M1 = 42.10 ± 11.90; M2 = 34.21 ± 13.94; *p* = 0.037, *d =* −0.609) dimensions, all with moderate effect sizes. These results indicate a decrease in emotional exhaustion and professional fatigue following the intervention, reflecting a positive effect of workplace exercise on the participants’ psychosocial balance. The client-related dimension did not show significant changes (*p* = 0.270), which may be explained by several factors. These include the short duration of the intervention, the limited variability and predominantly task-oriented nature of patient interactions in radiologic practice, and the fact that the exercise sessions were performed mainly on an individual basis. Together, these aspects may have reduced the potential for the intervention to influence interpersonal or relational components of burnout linked to patient contact.

Regarding perceived stress (PSS), a statistically significant reduction in mean values was observed in the experimental group, decreasing from 18.60 at M1 to 14.50 at M2 (*p* = 0.013; *d* = −0.697 (moderate)), indicating a lower perception of stress after participation in the workplace exercise program. Conversely, in the control group, the differences between M1 and M2 were not statistically significant for any of the evaluated variables. Quality of life remained virtually unchanged, with minimal variations and *p*-values above 0.05 across all domains. Similarly, burnout and perceived stress levels exhibited small, nonsignificant fluctuations, suggesting stability over time in the absence of intervention.

**Figure 2:**
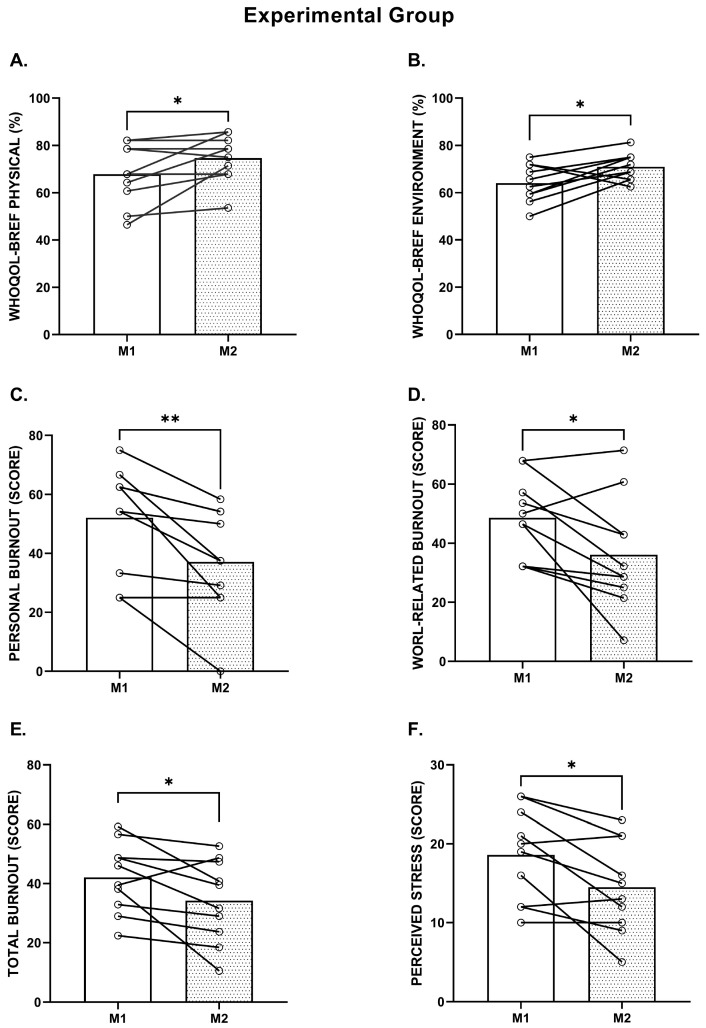
Differences between baseline (M1) and after 6 weeks of follow-up (M2) in the experimental group, in the domains (**A**) Physical and (**B**) Environment of the WHOQOL-BREF, as well as in (**C**) Personal Burnout, (**D**) Work-related Burnout, (**E**) Total Burnout, and (**F**) Perceived Stress Scale. The bar graphs represent the averages ± DP. * Indicates a statistically significant difference between time points (*p* < 0.05); ** *p* < 0.01.

To provide a more precise analysis and understanding of results for each domain, Figure 2 presents six graphs that illustrate the differences between the baseline (M1) and the six-week follow-up (M2) in the experimental group.

## 4. Discussion

This study aimed to assess the effects of a structured workplace exercise program on perceived stress, burnout, and quality of life among radiologic technologists. Overall, the main findings indicate that participants in the intervention group experienced improvements in selected quality-of-life domains (i.e., physical and environmental) and reductions in burnout (i.e., personal, work-related, and total burnout) and perceived stress levels. In contrast, the control group showed no meaningful changes between the two assessment points. These findings suggest that integrating brief, structured physical activity sessions into the workday may be a feasible approach to supporting psychosocial well-being in clinical settings; however, the results should be interpreted with caution due to the small sample size.

The relevance of these findings is reinforced by evidence showing that radiographers and radiologic technologists are exposed to substantial occupational demands and report high levels of burnout and job strain [1,14]. In broader healthcare contexts, burnout has been consistently linked to reduced well-being and work performance [33], highlighting the need for feasible preventive strategies within clinical environments.

The observed improvements in physical and environmental domains of quality of life are consistent with evidence from recent workplace exercise and active-break interventions among healthcare professionals [12,16,27]. Short, structured programs delivered during working hours (particularly those emphasizing stretching, mobility, and low-load strengthening) have been associated with improvements in physical comfort, perceived functioning, and work-related well-being [12,27]. Similarly, short-term exercise interventions in healthcare workers have shown beneficial effects on psychological outcomes and perceived well-being, supporting the plausibility of improvements in quality-of-life domains even in demanding settings [16]. For radiologic technologists specifically, these benefits may be partially explained by reduced musculoskeletal discomfort associated with prolonged static postures and repetitive tasks commonly encountered in imaging work [1,12,34].

Reductions in personal and work-related burnout, together with lower perceived stress, align with a growing body of literature identifying physical activity as a protective factor against occupational strain. A systematic review has highlighted physical activity as a potential protective factor against burnout symptoms and evidence among healthcare workers suggests that exercise may improve mood regulation and stress management [35]. In addition to physiological pathways, workplace-based activity may support recovery processes by creating structured opportunities for brief disengagement and recuperation during the workday, which is consistent with theoretical models of recovery and psychological detachment [36].

Including weekly reminders and illustrated handouts may have contributed to maintaining participants motivated and highly engaged throughout the intervention, representing a behavioral-support component that extends beyond the physical exercise itself. This supportive element may partly explain the positive results observed, as adherence and sustained engagement are key factors in the success of workplace health interventions. Therefore, adding simple behavioral strategies to reinforce participation should be considered in future workplace exercise programs [36].

Regarding client-related burnout, no significant change was observed after the intervention. This finding may reflect the specific nature of radiologic technologists’ patient interactions, which are often brief, task-oriented, and standardized, potentially limiting variability in patient-related emotional demands during routine practice. Prior work suggests that patient/client-related exhaustion is more strongly influenced by interpersonal and organizational factors (e.g., communication demands, workflow organization, perceived recognition) than by individual-level coping strategies alone [15]. As such, changes in this burnout dimension may require more extended intervention periods or multicomponent approaches that combine individual health promotion with organizational and relational components.

Despite the relevance of the findings, some limitations should be acknowledged. First, the small sample size limits the generalizability of the results and may have constrained statistical significance in specific domains. Second, the relatively short duration of the intervention may not have been sufficient to induce meaningful changes in social relationships or other structural aspects of well-being. Third, physical activity levels were not measured or controlled during the intervention. However, participants were instructed to maintain their usual lifestyle, including physical activity and dietary patterns; changes in these variables cannot be excluded.

Additionally, participation in a workplace intervention may have led to behavioral changes, such as increased attention or awareness (the Hawthorne effect). Finally, the use of self-reported measures, combined with the lack of participant blinding, may have increased the risk of response bias. Such limitations are common in small-sample pilot trials and underscore that the present findings should be interpreted as exploratory in nature [37].

Despite these limitations, the present study demonstrates that a brief, low-cost, and easily implementable workplace exercise program is feasible within a radiology department and can achieve high adherence. Similar feasibility has been reported in recent workplace-based interventions in clinical environments [12,16], supporting the practicality of embedding short exercise sessions into routine workflow. Future research should replicate these findings in larger samples, incorporate longer follow-up to assess sustainability, and include objective measures of physical activity and workload to clarify mechanisms and strengthen causal inference.

## 5. Conclusions

This study explored the potential impact of a structured workplace exercise program on perceived stress, burnout, and quality of life among radiologic technologists. No statistically significant between-group differences were observed. After six weeks of intervention, within-group reductions in perceived stress and in both personal and work-related dimensions of burnout were observed in the experimental group, along with improvements in the physical and environmental domains of quality of life. Effect sizes ranged from small to large across several outcomes, supporting the feasibility of integrating brief exercise sessions into the workday.

However, given the small sample size and the absence of significant between-group effects, these findings should be interpreted with caution and considered exploratory rather than confirmatory. Within these limitations, the study provides preliminary support for further investigation of workplace exercise programs. Future studies with larger, adequately powered samples and longer follow-up periods are needed to clarify the magnitude of potential effects.

## Figures and Tables

**Table 1:** Weekly Exercise Program Schedule.

	Weeks		
	1–2	3–4	5–6
Flexibility Training			
Frequency of training (times/week)	2×/day 3×/week	2×/day 3×/week	2×/day 3×/week
sets	1	2	2
Duration (s)	10	10	15
Auxiliary Exercise Equipment	No Equipment		
Balance Training—Static Exercises			
Frequency of training (times/week)	2×/day 3×/week	2×/day 3×/week	2×/day 3×/week
sets	1	2	2
Repetitions	10	12	15
Auxiliary Exercise Equipment	No Equipment		
Balance Training—Dynamic Exercises			
Frequency of training (times/week)	2×/day 3×/week	2×/day 3×/week	2×/day 3×/week
sets	1	2	2
Distance (m)	3	5	6
Auxiliary Exercise Equipment	Chair		
Strength Training			
Frequency of training (times/week)	2×/day 3×/week	2×/day 3×/week	2×/day 3×/week
sets	1	2	2
Repetitions	10	12	15
Auxiliary Exercise Equipment	Elastic Band/loop band		

**Table 2:** Baseline characteristics of participants by randomized group.

Variables	All (*n* = 19)	Control Group (*n* = 9)	Exercise Group (*n* = 10)	*p*-Value
Age, years	45.89 ± 9.27	48.22 ± 7.86	43.80 ± 9.92	0.326
Women, *n* (%)	12 (63.2)	6 (66.7)	6 (60.0)	1.000
Smoker, *n* (%)	2 (10.5)	2 (22.2)	0 (0.0)	0.211
Body Mass Index (kg/m^2^)	24.93 ± 3.50	24.33 ± 2.71	25.47 ± 4.10	0.491
Professional experience (years)	24.89 ± 9.52	27.22 ± 8.33	22.80 ± 10.45	0.326

Notes: Values are presented as mean ± standard deviation, or count (percentage) as appropriate. Abbreviations: *n*, number.

**Table 3:** Descriptive and inferential statistics of comparisons between moments in the experimental group.

	Domains	*n*	Min	Max	Mean	Standard Deviation	Normality	*p*	Eta Squared (η^2^)	*Effect Size d Cohen (CC 95%)*
WHOQOL	General (M1)	10	37.50	75.00	65.00	14.191	0.003	0.238 ^b^	0.139	0.804 (moderate)
	General (M2)	10	50.00	100.00	71.25	14.494	0.033			
	Physical (M1)	10	46.43	82.14	67.86	12.821	0.265	0.046 ^a^ *		0.592 (−0.303–1.488) (small)
	Physical (M2)	10	53.57	85.71	74.64	9.880	0.337			
	Psychological (M1)	10	45.83	70.83	60.00	8.146	0.394	0.062 ^b^	0.348	1.462 (large)
	Psychological (M2)	10	58.33	66.67	62.92	3.651	0.017			
	Social Relations (M1)	10	33.33	83.33	67.50	14.933	0.010	0.719 ^b^	0.013	0.229 (small)
	Social Relations (M2)	10	41.67	83.33	65.83	13.292	0.294			
	Environment (M1)	10	50.00	75.00	64.07	7.968	0.804	0.032 ^a^ *		0.991 (0.062–1.92) (moderate)
	Environment (M2)	10	62.50	81.25	70.94	5.714	0.713			
Burnout	Personal (M1)	10	25.00	75.00	52.08	17.922	0.087	0.004 ^a^ *		−0.834 (−1.748–0.08) (moderate)
	Personal (M2)	10	0.00	58.33	37.08	18.050	0.337			
	Work-related (M1)	10	32.14	67.86	48.57	13.596	0.201	0.026 ^a^ *		−0.756 (−1.664–0.151) (moderate)
	Work-related (M2)	10	7.14	71.43	36.07	19.007	0.650			
	Customer-related (M1)	10	4.17	54.17	24.58	15.522	0.253	0.270 ^b^		0.327 (−0.0556–1.09) (small)
	Customer-related (M2)	10	0.00	45.83	29.17	12.422	0.066			
	Total burnout (M1)	10	22.37	59.21	42.10	11.898	0.896	0.037 ^a^ *		−0.609 (−1.505–0.288) (moderate)
	Total burnout (M2)	10	10.53	52.63	34.21	13.938	0.779			
Perceived Stress	PSS score (M1)	10	10.00	26.00	18.60	5.910	0.361	0.013 ^a^ *		−0.697 (−1.6–0.206) (moderate)
	PSS score (M2)	10	5.00	23.00	14.50	5.855	0.779			

Notes: ^a^ Paired *t*-test, ^b^ Wilcoxon. * *p* ≤ 0.05—level of significance. Bold *p* values mean significant differences.

**Table 4:** Descriptive and inferential statistics of comparisons between moments in the control group.

	Domains	*n*	Min	Max	Mean	Standard Deviation	Normality	*p*	Eta Squared (η^2^)	*Effect Size d Cohen (CC 95%)*
WHOQOL	General (M1)	9	50.00	87.50	72.22	12.15	0.273	1.00 ^a^		<0.001 (−0.924–0.924) (trivial)
	General (M2)	9	50.00	87.50	72.22	13.66	0.172			
	Physical (M1)	9	39.29	100.00	67.86	16.66	0.816	0.836 ^a^		0.049 (−0.875–0.973) (trivial)
	Physical (M2)	9	53.57	96.43	68.65	15.34	0.059			
	Psychological (M1)	9	50.00	83.33	63.43	10.16	0.612	0.594 ^a^		0.093 (−0.831–1.018) (trivial)
	Psychological (M2)	9	50.00	79.17	64.35	9.57	0.963			
	Social Relations (M1)	9	58.33	100.00	75.93	14.10	0.575	0.376 ^a^		−0.285 (−1.214–0.643) (small)
	Social Relations (M2)	9	58.33	91.67	72.22	11.79	0.113			
	Environment (M1)	9	53.13	78.13	70.49	8.57	0.021	0.558 ^b^	0.034	0.377 (small)
	Environment (M2)	9	56.25	81.25	71.53	7.73	0.237			
Burnout	Personal (M1)	9	25.00	70.83	51.39	13.66	0.783	0.203 ^b^	0.162	0.879 (moderate)
	Personal (M2)	9	16.67	62.50	47.22	17.68	0.039			
	Work-related (M1)	9	21.43	57.14	42.06	12.97	0.310	0.247 ^a^		−0.363 (−1.295–0.569) (small)
	Work-related (M2)	9	14.29	57.14	36.90	15.36	0.559			
	Customer-related (M1)	9	4.17	41.67	17.13	13.41	0.022	0.507 ^b^	0.044	0.429 (small)
	Customer-related (M2)	9	0.00	54.17	15.74	17.28	0.015			
	Total burnout (M1)	9	26.32	52.63	37.14	8.62	0.707	0.278 ^a^		−0.325 (−1.255–0.605) (small)
	Total burnout (M2)	9	13.16	57.89	33.48	13.37	0.835			
Perceived Stress	PSS score (M1)	9	11.00	26.00	18.11	4.91	0.803	0.735 ^a^		0.114 (−0.811–1.039) (trivial)
	PSS score (M2)	9	9.00	29.00	18.78	6.69	0.937			

Notes: ^a^ Paired *t*-test, ^b^ Wilcoxon.

## Data Availability

The data presented in this study are available on request from the corresponding author due to ethical reasons.

## Supplementary Materials

The following supporting information can be downloaded at https://www.mdpi.com/article/10.3390/healthcare14081063/s1, Table S1: Exercise Program.

## Abbreviations

The following abbreviations are used in this manuscript:

ULSCB	Local Health Unit of Castelo Branco
CBI	Copenhagen Burnout Inventory
PSS	Perceived Stress Scale
M1	Baseline
M2	Follow-up
