# Supplementary material for: Effects of a Workplace Exercise Program on Stress, Burnout, and Quality of Life in Radiologic Technologists: A Randomized Controlled Trial
Source: Healthcare (Basel). 2026 Apr 16;14(8):1063. doi: 10.3390/healthcare14081063 (PMC13116276; doi:10.3390/healthcare14081063)
Supplement: Supplementary file 1 [file healthcare-14-01063-s001.zip › healthcare-4016653-supplementary.pdf]

Exercise	Description
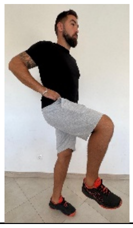	March in place for 15 seconds<br>Lift your knees to a comfortable level<br>Swing your arms or lean against a wall if necessary
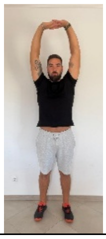	Clasp your hands over your head<br>Stretches your arms upward
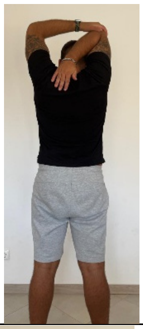	Raise one arm over your head<br>Bend the elbow and gently pull it with the other hand<br>Repeat with the other arm
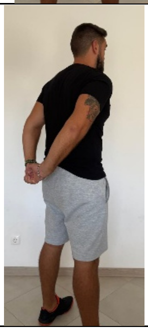	Clasp your hands behind your back.<br>Gently pull your arms back until you feel a stretch in the front of your shoulders and chest.
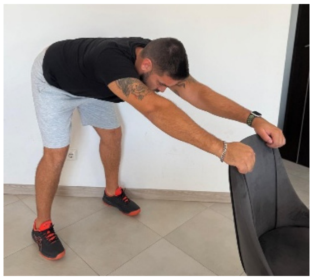	Rest your hands on the back of a chair.<br>Keep your trunk parallel to the ground.<br>Keep your legs slightly bent.<br>Slowly lower your chest to a comfortable limit.<br>Engage your abdominal muscles.

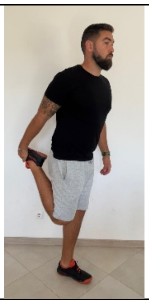

Lean against a wall or chair for support.
Hold your ankle and gently pull your foot toward your glutes.
Repeat with the other leg

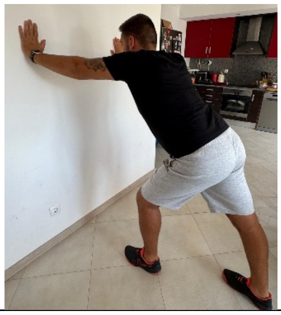

Place your hands against a wall.
Bend one leg and keep the other leg extended behind you.
Keep the back heel on the ground.
Repeat with the other leg

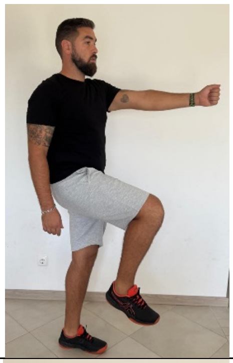

Lift and bend your knee to a comfortable position and maintain your balance
With the opposite hand, pretend to catch a ball from a teammate. Then switch and repeat with the other leg

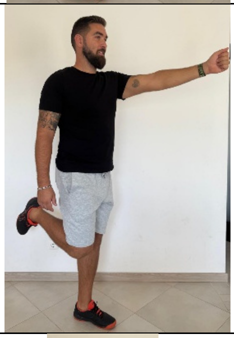

Bend your knee and bring your heel back toward a comfortable position while maintaining your balance
With the opposite hand, pretend to catch a ball from a teammate. Then switch and repeat with the other leg

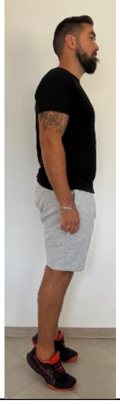

Rise onto your tiptoes
Maintain your balance

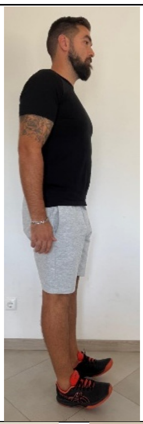

Place your heels down
Maintain your balance

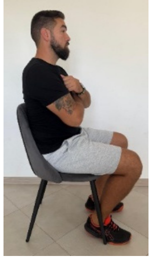 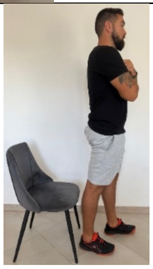

Sit in a chair
Cross your arms over your chest
Stand up and walk

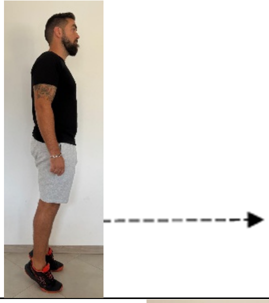

Rise onto your tiptoes
Move forward

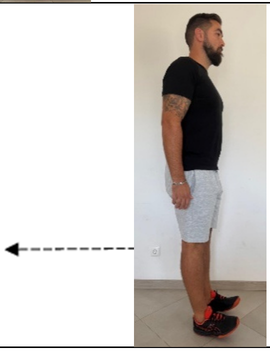

Place your heels down
Walk backward

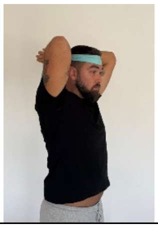

Place the band around your head
Hold the ends of the band with your hands
Stretch and bring your arms in, keeping the band taut

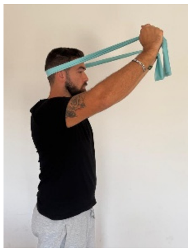

Place the band around your head
Hold the ends of the band with your hands
Extend and bring your arms in while keeping the band taut

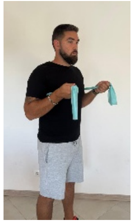

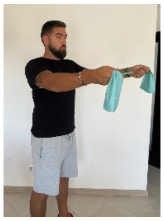

Keep your torso upright
Place the elastic band behind your back, under your arms
Extend and bring your arms in while keeping the band taut

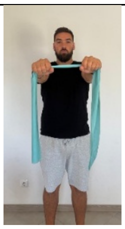

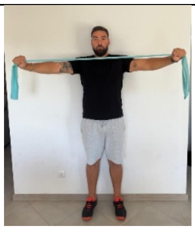

Extend your arms forward at shoulder height
Open your arms out to the sides while keeping them at shoulder height

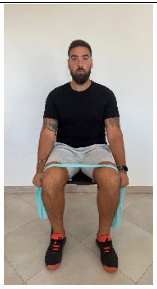

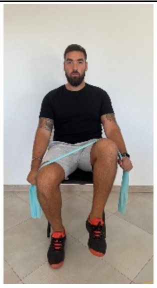

Sit on the edge of a chair
Keep your torso aligned
Lift one knee at a time

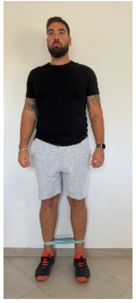

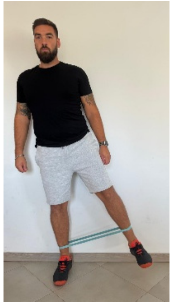

Open and close your leg while keeping the elastic band under tension
Repeat with the other leg
